# Evasion of the Interferon-Mediated Antiviral Response by Filoviruses

**DOI:** 10.3390/v2010262

**Published:** 2010-01-21

**Authors:** Washington B. Cárdenas

**Affiliations:** Laboratorio de Biomedicina, FIMCM, Escuela Superior Politécnica del Litoral (ESPOL), Campus Gustavo Galindo, Km 30.5 via Perimetral, Apartado 09-01-5863, Guayaquil, Ecuador; E-Mail: wbcarden@espol.edu.ec; Tel.: +5934 226 9589; Fax: +5934 285 0663

**Keywords:** IFN, VP35, VP24, filovirus

## Abstract

The members of the filoviruses are recognized as some of the most lethal viruses affecting human and non-human primates. The only two genera of the *Filoviridae* family, Marburg virus (MARV) and Ebola virus (EBOV), comprise the main etiologic agents of severe hemorrhagic fever outbreaks in central Africa, with case fatality rates ranging from 25 to 90%. Fatal outcomes have been associated with a late and dysregulated immune response to infection, very likely due to the virus targeting key host immune cells, such as macrophages and dendritic cells (DCs) that are necessary to mediate effective innate and adaptive immune responses. Despite major progress in the development of vaccine candidates for filovirus infections, a licensed vaccine or therapy for human use is still not available. During the last ten years, important progress has been made in understanding the molecular mechanisms of filovirus pathogenesis. Several lines of evidence implicate the impairment of the host interferon (IFN) antiviral innate immune response by MARV or EBOV as an important determinant of virulence. *In vitro* and *in vivo* experimental infections with recombinant Zaire Ebola virus (ZEBOV), the best characterized filovirus, demonstrated that the viral protein VP35 plays a key role in inhibiting the production of IFN-α/β. Further, the action of VP35 is synergized by the inhibition of cellular responses to IFN-α/β by the minor matrix viral protein VP24. The dual action of these viral proteins may contribute to an efficient initial virus replication and dissemination in the host. Noticeably, the analogous function of these viral proteins in MARV has not been reported. Because the IFN response is a major component of the innate immune response to virus infection, this chapter reviews recent findings on the molecular mechanisms of IFN-mediated antiviral evasion by filovirus infection.

## 1. Introduction to Filovirus

**Figure 1.**
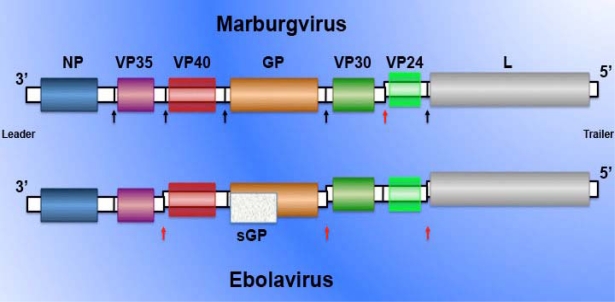
MARV and EBOV genome organization. Black and red arrows indicate intergenic regions. Red arrows depict stop transcription site of an upstream gene (genomic sense) overlapping the start transcription site of a downstream gene. sGP: Non-structural soluble glycoprotein, product of a non-edited GP gene transcript in EBOV species. Adapted from reference [1].

The filoviruses are members of the Mononegavirales with a non-segmented, negative-sense, single stranded RNA genome [1]. The *Filoviridae*, together with the Mononegavirales families *Paramyxoviridae* and *Rhabdoviridae*, replicate in the cytoplasm of infected cells and have a similar gene order that implies homologous function [2]. The only two genera in the *Filoviridae*, MARV and EBOV, contain species that cause severe hemorrhagic fever (HF) in human and non-human primates with case fatalities rates of 25 to 90% (Table 1). The genome of filoviruses is approximately 19,000 bases and has seven genes, which are arranged sequentially from the 3′ leader as follows: NP (nucleoprotein), VP35 (phosphoprotein), VP40 (matrix protein), GP (spike glycoprotein), VP30 (minor nucleocapsid), VP24 (minor matrix protein) and L (RNA-dependent RNA polymerase). In EBOV species, a transcriptional editing event that results in the insertion of an additional adenosine is required for viral GP expression. Non-edited GP transcripts result in the expression of the soluble non-structural GP protein (Figure 1).

The outbreak of a severe HF among vaccine plant workers in Germany and former Yugoslavia lead to the identification of the first filovirus species, the MARV, in 1967 [3]. It is likely that MARV was already circulating in the African primate population before it was imported into the western laboratories unaware of its existence. The second filovirus infection, but the first in a natural setting, was reported almost 10 years later in two nearly simultaneous outbreaks in southern Sudan and Zaire (the present Democratic Republic of Congo, DRC). These outbreaks led to the discovery of the second genus of the *Filoviridae* family, EBOV, and the two new species of the genus, Sudan Ebola virus (SEBOV) and ZEBOV, respectively [4,5].

The third species of EBOV was identified during another inadvertent importation of infected macaques from the Philippines into a quarantine facility in Reston, Virginia, USA, in 1989 [23]. This novel virus, named Reston Ebola virus (REBOV), was able to infect humans, as assessed by serology, but without apparent severe illness. In 1994, in Côte d’Ivoire, west-central Africa, after conducting the necropsy on a wild chimpanzee, a female researcher became ill with symptoms that included high fever, headaches, myalgia, cough, abdominal pain, diarrhea, vomiting, and macular rash [32]. The patient recovered and the fourth novel species of EBOV, Côte d’Ivoire Ebola virus (CIEBOV), was isolated from her blood on days 4 and 8 after the onset of the symptoms. Reports of a developing outbreak of HF in western Uganda, Bundibugyo district, in November 2007, lead to the identification of the fifth EBOV species, Bundibugyo Ebola virus (BEBOV) [31]. The initial serological identification of an EBOV as the etiology agent for the outbreak failed to be corroborated by the more sensitive real time reverse transcriptase-polymerase chain reaction (RRT-PCR) diagnostics. Sequencing of an amplicon obtained with a primer set that targeted filovirus L-gene showed sequence differences from known MARV or EBOV that explained the negative results obtained previously.

Since the first outbreak of ZEBOV, identification of the natural reservoir of filovirus proved to be elusive [33]. Recently, the development of more sensitive immunochemicals and molecular genetic diagnostics has permitted the detection of filovirus antigens, immunoglobulin G, and viral nucleic acids in at least four species of fruit bats [34,35]. Experimental infections revealed that fruit and insectivorous bats were able to support viral replication without apparent signs of illness when inoculated with ZEBOV [36]. Additionally, Towner *et al*. successfully isolated, for the first time, MARV from a cave-dwelling fruit bat (*Rousettus aegyptiacus*), possibly solving the long-standing enigma of the filovirus natural reservoir [37]. A recent outbreak of REBOV in pigs in the Philippines showed, for the first time, that Ebola HF can occur in a non-primate host [38]. These findings complicate the picture regarding the role that pigs may have in the chain of transmission of filovirus to human and non-human primates.

## 2. Filovirus pathogenesis

All reported symptomatic filovirus infections result in a severe HF in humans, non-human primates, and, as recently discovered, also in domestic pigs [38,39]. Although recognized human filovirus HF outbreaks have not reached pandemic proportions since first recognized in 1967, filoviruses represent a major public health concern for the following reasons: lethality, increase in outbreak episodes, emergence of new strains, lack of an approved vaccine, and potential development as a bio-weapon. The viral HF caused by members of the *Filoviridae* family, as well as members of *Arenaviridae* and *Bunyaviridae*, occurs largely in developing countries where detailed epidemiological and immunological accounts of disease onset are difficult to obtain [40]. Additionally, the high level of biological containment required to work with these viruses presents a major obstacle in the understanding of the pathogenesis of filovirus HF in humans. Despite these difficulties, several studies have gathered key clinical and immunological data on natural human infections of EBOV. The clinical signs of Ebola HF (EHF) include a combination of symptoms such as fever, abdominal pain, asthenia, diarrhea, headaches, arthralgia, and myalgia [13,41]. Among these, bilateral conjunctival injection, maculopapular rash and sore throat, with odynophagia, are suggestive of EHF [41]. Poor prognosis for the disease is associated with bleeding of the mucosa, anuria, hiccups, and tachypnea that lead to a state of stupor with polypnea and renal failure before death [41,42]. Further analysis of the humoral responses to EBOV infection in patients that succumbed or survived in an outbreak setting generated a unique set of data that helped our understanding of EHF pathogenesis. The initial study that characterized serum levels of cytokines in EBOV-infected patients showed increased levels of IL-2, IL-10, TNF-α, IFN-γ, and IFN-α associated with fatal outcomes of EHF compared with survivors or non-infected control samples [42]. A closer look at the immune response to EBOV infection showed that survivorship was associated with timely production of pro-inflammatory cytokines, anti-inflammatory cytokines, and activation of T cells [43,44]. An inverse relationship was observed between the levels of viral antigen and virus-specific IgGs in the plasma of EBOV-infected patients. Early appearance of specific IgG antisera, or lack of it, characterized survival *versus* fatal outcomes, respectively [43,44]. Interestingly, antisera of symptomatic and asymptomatic EBOV infected patients were directed largely against the viral NP, followed by the matrix protein, and then the phosphoprotein [43,45]. IgG against the viral spike glycoprotein was not detected. Nevertheless, promising recombinant vaccine vectors against filoviruses are based mainly on the expression of the viral surface glycoprotein [46,47]. Although the presence of specific antisera is a good prognosis for EHF patients, protection from EBOV in experimentally infected model animals, assessed by passive transfer of heterologous hyperimmune serum or with monoclonal antibodies, has given mixed results [48–51]. When protection was attained in these experiments, anti-serum was administered before or soon after infection. These results imply that other humoral and cellular factors triggered during the initial infection are key to determining the disease outcome in nature. In agreement with this assumption, an experimental whole blood transfusion from convalescent EHF patients conferred protection to seven out of eight symptomatic-phase EHF patients [52]. However, a recent experiment in a non-human primate model for EHF showed that whole blood transfusion from a convalescent-phase EHF monkey did not protect naïve monkeys when challenged with EBOV [53]. This last study underscores the importance of an effective innate immune response to infection; it will set the tone for a balanced production of stimulatory and inhibitory signals, leading to a specific cellular control of viral infection. This notion agrees with studies of inflammatory responses to EBOV infection in symptomatic survivors and asymptomatic cases. In these studies, a common pattern of early (within a week or so from infection) production of pro-inflammatory cytokines and chemokines, such as IL-1β, IL-6, TNF-α, MIP-1α, MIP-1β and MCP-1, was followed by increasing levels of the anti-inflammatory molecules IL-1RA, sTNF-RI, sTNF-RII, and IL-10 and the T cell activation markers CD28, CD40L, CTLA4, Fas, FasL, and perforin [43–45,54]. This chain of immunological events is likely disrupted in fatal cases of filovirus HF by MARV and EBOV targeting host dendritic cells, which are key antigen presenting cells (APC) that modulate innate and adaptive immune responses [55–57]. Indeed, a delayed adaptive immune response, as assessed by the formation of CD8^+^ specific T cells, was observed in a lethal mouse model of EBOV-infection. Although these EBOV-specific CD8^+^ T cells did not protect infected animals, they underwent expansion when adoptively transferred into EBOV-challenged naïve mice; these mice were protected [58]. Filovirus infection may affect signal integration through the receptor-like protein tyrosine phosphatase CD45, an important regulator of signaling thresholds in immune cells [59,60]. Additionally, innate antiviral cellular responses, mediated by natural killer (NK) cells, were also impaired in a mouse model of EBOV infection [61]. Activation of NK cells, as assessed by cytokine production and cytolysis of suitable targets including filovirus-infected dendritic cells (DCs), was only attained by filovirus-derived virus-like particles (VLPs). Exposure to live or inactivated filovirus did not stimulate the antiviral effects of NK cells [61,62]. Taken together, these data reinforce the view of an early disruption of host innate immune responses during filovirus infection.

## 3. Evasion of interferon response by Filovirus

The molecular mechanisms of viral pathogenesis are poorly understood for most members of the family *Filoviridae*. During the last ten years, a steady increase in filovirus research has unveiled important virulence factors and signaling pathways that may explain the immunosuppressive characteristic of filovirus infection. Several potential mechanisms contributing to filovirus virulence have been reviewed [39,63]. These mechanisms include cytotoxicity of the viral GP, the production of pro-inflammatory cytokines, and the dysregulation of the coagulation cascade due to the production of tissue factor [64–68]. Each of these processes, however, likely occurs as a result of active replication of the virus. Thus, the ability of the virus to counteract early antiviral responses likely plays an important role in virulence of ZEBOV, the best characterized filovirus [39]. At the center stage of the cellular antiviral innate immune response are the IFN-α/β cytokines [69,70]. IFN-α/β are multifunctional cytokines that regulate the innate and adaptive immune responses by affecting, among other things, the function of key immune cells like DCs [71]. Several studies have demonstrated the ability of EBOV infection to block cellular responses to IFN [72–74] and the IFN system plays a role in preventing EBOV disease in mice [75]. Because the activation of the IFN system is a central component of the host response to viral infection, it is not surprising that EBOV has evolved mechanisms to evade its activation. Indeed, the EBOV protein VP35, which also functions as a viral polymerase co-factor and a structural protein, has IFN antagonist activity [55,76,77]. VP35 was initially identified as an IFN-antagonist protein because ectopic expression of it rescued the impaired growth of an influence A virus mutant lacking the interferon antagonist protein NS1 (delta NS1 virus) [77]. Furthermore, ectopic expression of VP35 inhibited activation of an interferon stimulated response element (ISRE)-containing promoter when either transfected dsRNA or viral infection was used as the activating stimulus [77].

A more detailed study of the mechanism by which VP35 influenced the host IFN response showed that the initial steps of IFN production, and not IFN signaling from the IFN-α/β receptor, were impaired by VP35 [76]. The ability of VP35 to block IFN-α/β production correlated with its ability to inhibit the phosphorylation, and thus activation of, interferon regulatory factor 3 (IRF-3) [76]. SUMOylation of IRF-7 mediated by VP35 was recently described as an additional mechanism of repression of transcription of IFN genes [78]. IRF-3 and IRF-7 are transcription factors at the center of the cellular antiviral program [79,80]. IRF-3 is constitutively expressed in many tissues and is located in the cytoplasm of unstimulated cells [81,82]. IRF-7 is expressed at low levels in somatic cells and DCs, but at high levels in plasmacytoid dendritic cells (pDCs) [83]. Upon viral infection, IRF-3 and IRF-7 are phosphorylated at their carboxy-termini (C-terminus), which leads to dimerization, nuclear translocation, and association with other trans-activator proteins. Activated IRF-3 triggers the expression of IFN-α/β and IFN-α/β-inducible genes, leading to the establishment of an antiviral state [81,84]. Signal amplification is attained by an IFN feedback loop that upregulates IRF-7 expression and activation of IFN-α genes [79]. Activation of IRF-3 and IRF-7 requires the non-canonical kinases IKKɛ and TBK-1 [85–87]. In pDCs, activation of IRF-7 requires IL-1 receptor-associated kinase (IRAK)-1 [88]. Interestingly, EBOV VP35 protein was shown to interact with IKKɛ and TBK-1, implying a mechanism for its IFN antagonist function [89]. Further, an *in vitro* kinase assay showed that VP35 was a substrate for IKKɛ and TBK-1, and increasing ectopic expression of VP35 in cells transfected with IKKɛ resulted in reduction of phosphorylation of a C-terminus IRF-3 recombinant protein. Consistent with the kinase assay results, both VP35 and IRF-3 interacted with the IKKɛ kinase domain; additionally, increasing ectopic expression of VP35 was able to impair IRF-3-IKKɛ kinase domain interaction [89]. More important, preliminary data from the same group showed that IKKɛ, and to a lesser extent TBK-1, was able to phosphorylate VP35 in the context of a T7-driven minigenome system. Strikingly, minigenome reporter activity was enhanced by IKKɛ, but only a marginal effect was observed with TBK-1; these results correlate with the observed VP35 phosphorylation patterns (C. Basler, personal communication).

IKKɛ and TBK-1 are key components of the viral-activated kinases that lead to IRF-3 and IRF-7 activation [85,86]. Although TBK-1 is ubiquitously distributed, IKKɛ is expressed mainly in the thymus, spleen, and peripheral blood leukocytes [90,91]. TBK-1 deficient embryonic fibroblasts have impaired IFN-α/β responses to virus infection. However, residual IKKɛ may partially compensate for the loss of TBK-1. In contrast, TBK-1 was completely dispensable for IFN-α/β responses to virus infection in mouse bone marrow derived macrophage (BMM), where IKKɛ was predominant [87,92,93]. As mentioned before, DCs, together with monocytes/macrophages, are the primary targets for EBOV and MARV infections [55–57,67,74,94]. EBOV VP35 was shown to impair murine DC maturation induced by virus and lipopolysaccharide. VP35 prevented expression of surface markers and production of cytokines, including IFN-α/β, by DCs, which resulted in poor CD4^+^ T cell activation [95]. A useful strategy to establish infection in different host tissues can be to block the downstream signaling of the IRF kinases. Indeed, EBOV VP35 protein is able to block IFN-β reporter gene activation by TBK-1 or IKKɛ [96]. Thus, it seems that EBOV exploits VP35 as a decoy substrate for the kinases. It will be of interest to determine whether IKKɛ phosphorylation of VP35 modulates EBOV polymerase function.

The search for the IRF-3 inhibition domain of EBOV VP35 identified a basic amino acid-rich motif, similar to the N-terminal dsRNA-binding motif of the influenza A NS1 protein, which suggested an IFN-antagonist function based on the dsRNA-binding capability of VP35 [97]. Mutation of key residues in this region diminished the ability of VP35 to block viral-induced IFN-β production; however, these forms of VP35 maintained the ability to support viral replication as assessed by a minigenome system or by rescuing recombinant virus [98]. A closer look at the putative dsRNA-binding motif of the VP35 protein showed that VP35 was able to bind dsRNA-type molecules and that the individual mutations R312A and K309A abolished this activity [96]. Consistent with its dsRNA-binding properties, VP35 was shown to block protein kinase R (PKR) activation [99,100] and function as a RNA silencing suppressor [101]. The crystal structure of the C-terminus IFN inhibitory domain of VP35 revealed a fold consistent with dsRNA-binding [102]. However, assessment of the IFN-antagonist function of the VP35 R312A and K309A mutants revealed that these mutations retain an important inhibitory function, as measured by reporter gene activation with Sendai viral infection, retinoic acid-inducible gene I (RIG-I), and the CARD-containing mitochondrial protein IPS-I [96]. These data suggest there is an alternative mechanism for VP35 IFN-antagonist function.

**Figure 2.**
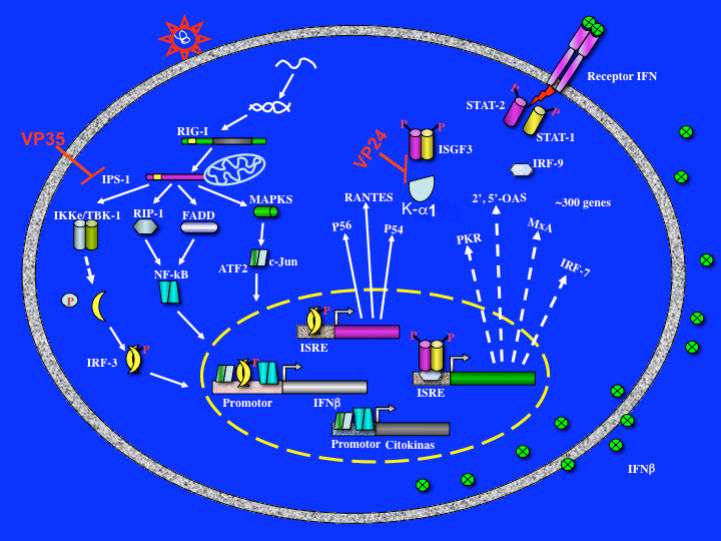
Model of ZEBOV VP35 and VP24 inhibition of host IFN-α/β responses.

The evidence suggests that EBOV VP35 protein targets IKKɛ and TBK-1 to control the innate immune response of the host. In agreement with this, VP35 was able to block reporter gene activation by the cytoplasmic viral RNA sensor RIG-I [103]. RIG-I, along with melanoma differentiation associated gene-5 (MDA-5) and the laboratory of genetics and physiology-2 (LGP2), belongs to a family of RIG-I like receptors (RLR) that function as cytoplasmatic sensors of viral RNA upstream of IRF-3 activation [104]. Ebola VP35 was also able to impair reporter gene activation by ectopic expression of IPS1/MAVS/VISA/Cardif and disrupted protein-protein interactions with IKKɛ [89,96,105–108]. These results imply that Ebola VP35 protein disrupts downstream signaling from IKKɛ and TBK-1 and upstream signalosome interactions (Figure 2).

Several signaling pathways have been shown to activate IRF-3 and induce IFN-α/β production. Toll-like receptors (TLR) 3 and 4 are pathogen associated molecular pattern receptors that can specifically lead to IRF-3 activation and trigger the transcription of genes involved in the defense against viral infection [109]. TLR3 is activated by poly (I:C), a synthetic dsRNA analog; bacterial lipopolysaccharide (LPS) is the ligand for TLR4 [110,111]. TLR signals are transduced to target genes through the interaction of the Toll-interleukin-1 receptor (TIR) domains found in the cytoplasmic tails of TLRs with TIR-containing adapter proteins. Currently, there are four TIR adapters with defined functions in mammalian TLR signaling: MyD88, TIRAP, TRIF, and TRAM [112,113]. Although MyD88 has been implicated in signaling by all TLRs, TRIF and TRAM are involved in MyD88-independent signaling by TLR3 and TLR4, leading to IRF-3 activation [114–117]. TRIF is involved in signaling to IRF-3 from both TLR3 and TLR4 [86,114,116]. TRAM, which interacts with TRIF, is required in addition to TRIF for TLR4-induced activation of IRF-3 [86,115]. A recent report showed that engagement of TLR4 by the EBOV GP on VLPs led to the secretion of pro-inflammatory cytokines and suppressor of cytokine signaling 1 (SOCS1) in a human monocytic cell line and HEK293 cells stably expressing a TLR4/MD2 complex [118]. Interestingly, SOCS1 was reported to regulate the IFN-dependent pathways by reducing IFN-β production and STAT1 phosphorylation [119]. Human DCs stimulated with EBOV VLPs induced the activation of NF-κB and ERK1/2 signaling pathways, resulting in the production of several pro-inflammatory cytokines [120].

**Figure 3.**
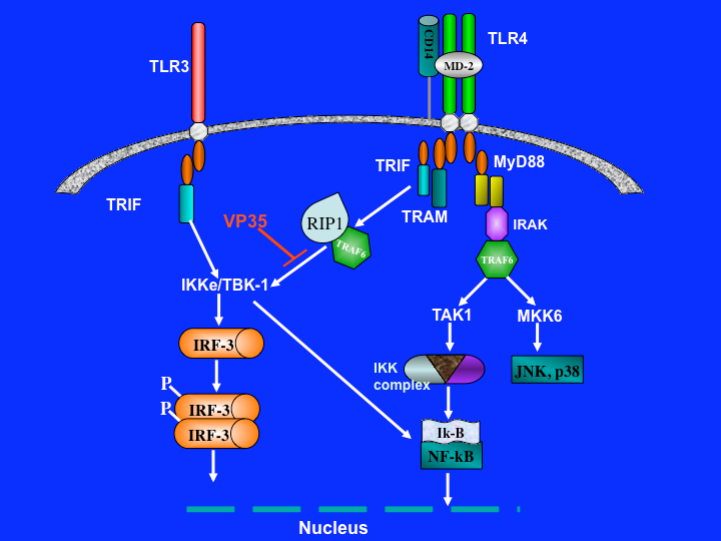
Ebola VP35 blocks TRIF and TRAM signaling arm of TLR4.

The observed pattern of activation was very similar to the pattern observed with LPS treatment, and thus likely to involve TLR4. It has also been reported that EBOV VP35 can inhibit the IRF-3-dependent reporter gene activation induced by ectopic expression of TRIF or TRAM in HEK293 cells [121]. Moreover, activation of reporter gene by the co-expression of TRIF and TRAM was greatly impaired by VP35. These results agree with the observation that ectopic expression of VP35 in U373 cells was able to block reporter gene activation induced by LPS (Figure 3). At this point, it is unclear what benefit EBOV gains by inhibiting the TRIF/TRAM arm of TLR4 signaling, but we can speculate that activation of signaling through TLR4 by EBOV GP can induce the production of pro-inflammatory cytokines and chemokines that contribute to EHF pathogenesis [118]. Viral replication can proceed, possibly by VP35 blocking the TRIF-TRAM arm of IFN-β activation, thus evading the antiviral function of this cytokine. Indeed, EBOV VP35 protein was shown to impair murine DCs maturation induced by LPS, an agonist of TLR4 [95]. TLR1 upregulation was observed in EBOV- and MARV-activated neutrophils, but the pathogenic importance of this event has not yet been established [122].

Recently, a new mechanism of ZEBOV IFN-antagonist function has been described. In addition to the well-known inhibition of IFN-β and α-4 production by ZEBOV VP35 protein, the minor matrix protein VP24 inhibits IFN-signaling through the JAK-STAT pathway [123]. Therefore, it seems that ZEBOV is well-equipped to antagonize both arms of the IFN antiviral innate immune response; this ability correlates with the high fatality rates associated with EHF outbreaks. A well-characterized set of experiments showed that the inhibition of IFN signaling mediated by VP24 was associated with the impediment of phosphorylated STAT-1 (PY-STAT1) movement to the nucleus. By specifically recruiting karyopherin α-1, VP24 blocked the interaction of PY-STAT-1 with this nuclear transport protein; karyopherin α-1 stayed in the cytoplasm, effectively inhibiting transcription of IFN-stimulated genes. Inhibition of PY-STAT1 nuclear translocation would impair both Type I and Type II IFN signaling. VP24 appears to compete with PY-STAT1 for the karyopherin α-1 C-terminus region [124]. Furthermore, VP24 proteins from mouse-adapted strains of ZEBOV and REBOV were also able to block PY-STAT1 nuclear translocation, suggesting a conserved mechanisms of VP24 IFN-antagonist function among EBOV species. Mutational analysis of VP24 identified two regions necessary for IFN-signaling antagonist function and karyopherin α-1 binding [125]. Individual amino acid changes at W42A or K142A resulted in VP24 partial loss of binding to karyopherin α-1 and in its ability to inhibit IFN-β-induced gene expression; however, generation of a W42A/K142A double mutant was necessary for at complete loss of binding to karyopherin α-1 and a strong reduction in its IFN-antagonist function [125]. Unfortunately, attempts to rescue a recombinant EBOV that incorporated diverse VP24 mutations were not possible.

The other member of the *Filoviridae*, MARV, provoked a general suppression of the antiviral immune response in hepatoblastoma cells treated with IFNα. As with members of the EBOV, host STAT-1 appears to be the target for the IFN-antagonist signaling function. But, contrary to EBOV, MARV appeared to inhibit STAT-1 and STAT-2 phosphorylation [126]. The last outbreak of MARV in Angola, Africa, clearly indicated that this filovirus can be as deadly as EBOV [9]. Despite the lethality of MARV, identification of the IFN-antagonist proteins analogous to the EBOV counterpart has not been reported yet.

## 4. Conclusions

Filovirus disease represents a public health concern. The last Angola outbreak of an unsuspected MARV underscored the importance of understanding the molecular mechanisms of filovirus pathogenesis. Despite the efforts that working with this Category A pathogen demand and the political and social constraints in gathering important clinical information in an outbreak setting, there is a good amount of high quality research that has already allowed us to think about protective vaccines and chemotherapeutic strategies. The parallel development of high impact research on the basic innate immune components, interactions, and modulators that are required to mount an effective antiviral response in mammals has also helped us to identify possible targets that may explain the mechanisms of immune suppression during filovirus infection. Additionally, productive collaboration among filovirus researchers has supported the rapid development of the field.

## Figures and Tables

**Table 1.** Documented Filovirus infections/outbreaks since 1967.

**Virus**	**No.**	**Location**	**Year**	**Human Cases (deaths)**	**CFR%**
Marburg	1	Germany (Marburg and Frankfurt), former Yugoslavia (Belgrade) [6].	1967	31 (7)	23
	2	South Africa (Johannesburg) [7].	1975	3 (1)	33
	3	Kenya (Mount Elgon National Park) [6].	1980	2 (1)	50
	4	Kenya (Mount Elgon National Park) [6].	1987	1 (1)	100
	5	DRC (Durba, gold mine village) [8].	1998–2000	154 (128)	83
	6	Angola (Uige Province) [9].	2004–2005	252 (227)	90
	7	Uganda (mine workers in Kakasi Forest Reserve, Kamwenge District) [6].	2007	3 (1)	50
	8	Uganda (western tourists at Maramagambo Forest) [10].	2008	2 (1)	50
Ebola-Zaire	1	DRC, formerly Zaire. (Yambuku and surroundings) [4].	1976	318 (280)	88
	2	DRC, formerly Zaire (Tandala Hospital, Tandala) [11].	1977	1 (1)	100
	3	Gabon (Makokou General Hospital and gold-panning encampment) [12].	1994/1995	49 (29)	59
	4	Gabon (outbreak began early February in the village of Mayibout 2, Gabon) [12].	1996	31 (21)	67.7
	5	Gabon (outbreak started in a logging camp near Booué) [12].	1996/1997	60 (45)	75
	6	DRC, formerly Zaire (outbreak centered in Kikwit and surrounding area) [13].	1995	315 (250)	79.4
	7	South Africa (Imported case from Libreville, Gabon. Single fatality was the local nurse caring for the index case) WHO [14].	1996	2 (1)	50
	8	Gabon and Republic of the Congo (Simultaneous outbreaks in La Zadié, Ivindo and Mpassa districts, Gabon, and Mbomo and Kéllé districts, Congo) [15].	2001/2002 (25 October to 18 March)	124 (97)	78
	9	Republic of the Congo (outbreak was in Mbomo district, Congo, where two fatal cases migrated to Ekata village in Gabon) [15].	2002 (17 May to 25 July)	11 (10)	91
	10	Republic of the Congo (outbreak was mainly present in the Kéllé district with fewer cases in the Mbomo district) [16].	2002/2003 (25 December to 22 April)	143 (128)	89.5
	11	Republic of the Congo (Outbreak affected the Mbomo and Mbandza villages of the Mbomo district) [17].	2003	35 (29)	83
	12	Republic of the Congo (outbreak was in the west part of the country, in the Cuvette Ouest Region, towns of Etoumbi and Mbomo) [18].	2005 (25 April to 16 June)	12 (9)	75
	13	Democratic Republic of Congo (outbreak was in Mueka & Luebo health zones, Province of Kasai Occidental. Reports started in September 2007 until official end declaration of the outbreak on 16 February 2009) [18].	2007/2009	32 (15)	47
Ebola-Sudan	1	Sudan (Towns of Nzara, Maridi and Tembura) [5].	1976	284 (151)	53
	2	England (Accidental laboratory inoculation) [19].	1976	1 (0)	0
	3	Sudan (Nzara and Yambio in Southern Sudan) [20]	1979	34 (22)	65
	4	Uganda (Outbreak initiated in the Gulu district, then spread to Mbarara and Masindi districts) [21].	2000/2001	425 (224)	52.7
	5	Sudan (outbreak occurred in Yambio county, southern Sudan) [22].	2004 (15 April to 26 June)	17 (7)	41
Ebola-Reston	1	USA (New EBOV in Reston, Texas, introduced with infected cynomolgus macaques from Philippines) [23].	1989	0 (0)	0
	2	USA (Pennsylvania, serologic evidence of infection in 4 animal handlers) [24].	1990	0 (0)	0
	3	Philippines (Ebola-like virus present at primates export facilities) [25].	1989/90	0 (0)	0
	4	Italy (Ebola-like virus causing hemorrhagic fever in Macaques imported from Philippines) [26].	1992	0 (0)	0
	5	USA (Outbreak in a Texas quarantine facility due to infected cynomolgus macaques imported from Philippines. Human seroconversion was not detected) [27].	1996	0 (0)	0
	6	Philippines (A single primate export facility in the island group of Luzon appeared to be the source of infected primates in the USA) [28].	1996	0 (0)	0
	7	Philippines (outbreak occurred in two farms located in Bulacan & Pangasinan provinces. First report of a filovirus infecting a non-primate mammal) [29].	2009	6 (0)	0
Ebola-Ivory Coast	1	Cote-d’Ivoire, central west Africa (a 39-year-old female was infected when she autopsied a dead chimpanzee) [30].	1994	1 (0)	0
Ebola-Bundibugyo	1	Uganda (outbreak occurred in Bundibugyo district, western Uganda. A new Ebola virus species was identified as the cause of the outbreak) [31].	2007/2008 (28 November to 20 February)	149 (37)	25

CFR=Case fatality rate.
